# The prevalence and incidence of HIV in the ART era (2006 – 2016) in North West Tanzania

**DOI:** 10.1177/09564624211065232

**Published:** 2022-01-18

**Authors:** Neema R. Mosha, Jim Todd, Crispin Mukerebe, Milly Marston, Soledad Colombe, Benjamin Clark, James Beard, Baltazar Mtenga, Emma Slaymaker, Ties Boerma, Basia Zaba, Mark Urassa

**Affiliations:** 1 Division of Epidemiology and Biostatistics, Faculty of Medicine and Health Sciences, Stellenbosch University, Cape Town-South Africa; 2 Mwanza Intervention Trials Unit, Mwanza, Tanzania; 3 National Institute for Medical Research, Mwanza Centre, Mwanza, Tanzania; 4 Dept of Population Health, London School of Hygiene and Tropical Medicine, London, UK; 5 Public Health Agency of Sweden, Solna, Sweden; 6 University of Manitoba, Winnipeg, MB, Canada

**Keywords:** HIV, ART, Epidemiology, AIDS

## Abstract

**Background:**

Sub-Saharan countries bears a disproportionate percentage of HIV infections and HIV related deaths despite the efforts to strengthen HIV prevention and treatments services, including ART. It is important to demonstrate how these services have contributed to reducing the epidemic using available population data.

**Methods:**

We estimated the prevalence and incidence rates from a cohort running over 23 years in Magu District, Mwanza Region-North West Tanzania. Adults 15 years and over who were residents of the Kisesa observational HIV cohort study between 2006 and 2016 were eligible for inclusion. Survival analysis was used to calculate person-time at risk, incidence rates and 95% confidence intervals (CI). Cox regression models were used for the risk factor analyses disaggregated by sex and age group.

**Results:**

The HIV prevalence in the sero-surveys decreased from 7.2% in 2006/07 to 6.6% in 2016, with a notable decrease of over 50% for both men and women aged 15-24 years. The incidence rate for HIV was estimated to be 5.5 (95% CI 4.6 - 6.6) per 1000 person-years in women compared to 4.6 (95% CI 3.5 – 5.8) in men, with a decrease over time. Despite the availability of ART services, the uptake is still small.

**Conclusions:**

New infections are still occurring, with high HIV incidence in individuals aged below 45 years. With new guidelines and the 95-95-95 UNAIDS target, prevalence and incidence must be adequately assessed. In addition, there is a need for additional efforts to assess the impact of HIV/AIDS prevention programmes and intervention services, especially in these areas where resources are limited.

## Introduction

Efforts to strengthen HIV prevention and treatment programmes have led to a reduction in the global incidence of HIV. Since 2000, the annual number of new HIV infections (all ages) has declined by 60%, from 2.8 to 1.7 million in 2018. Similar trends have been reported in sub-Saharan Africa (SSA), with declines of 47% in HIV incidence from an estimated 2.2 million cases in 2005 to 1.16 million cases in 2016 (1–6). With a significant decrease reported from some cohort studies in Zimbabwe (7), South Africa (8,9), Uganda (10) and Kenya(1).

However, SSA still bears a disproportionate percentage of HIV infections and HIV-related deaths, with an estimated 25.5 million people living with HIV and 730,000 deaths every year (11). Within the SSA, Southern and Eastern Africa bear a tremendous burden of HIV, with around 800,000 new infections and 310,000 deaths in 2018(12).

Estimates of the size and impact of the HIV epidemic can be made using routine data in countries with comprehensive vital registration and disease notification systems. However, in many low and middle-income countries (LMIC), these are absent or incomplete, and Demographics and Health Surveys (DHS) are used alongside sentinel surveillance in antenatal clinics to provide estimates of HIV prevalence(13). While prevalence has been used to assess epidemic patterns and trends (14–16), the incidence is a more useful indicator of the changing dynamics of disease transmission and provides a better estimate of the current state of the epidemic. Measurement of HIV incidence is more challenging and expensive as it relies heavily on prospective cohort studies, mathematical models and laboratory-based HIV incidence assays, with longitudinal studies providing the best estimates (13–15,17).

A longitudinal study in rural Tanzania by Mwita et al. provided the most recent estimate of HIV prevalence and incidence between 1994 and 2004 (18,19), which was before the rollout of ARV’s treatment in this area. In that period, there were minor increases in HIV prevalence from 6.0% (1994/95) to 6.7% (1996/97) in men, and stable estimates of 8.3% (1999/2000) to 8.2% (2003/04) in women. HIV incidence among adults aged 15-60 years was 12.6 per 1000 person-years between 1999 and 2004. Before 2017, these were the only population-based HIV incidence data for Tanzania. The Tanzania HIV Impact Survey (THIS), conducted during 2016/2017 as part of the Population-Based HIV Impact Survey (PHIA) Project, estimated a national annual incidence of 0.17% in men and 0.34% in women aged 15-64, with a significantly higher infection among adult aged 25-34 compared to other age groups. The report also showed women were five times more likely to become HIV positive compared to men(20), while The Tanzanian Demographic and Health Surveys (TDHS) have demonstrated the prevalence of HIV among adults aged 15-49 years has decreased from 7.0% in 2003/2004 (21) to 4.7% in 2017(20). HIV attributable adult mortality also reduced, from 110,000 in 2005 to 32,000 deaths in 2017(22).

ART services in Tanzania were initialised in October 2004 and were steadily decentralised from tertiary to primary health care facilities. ART services expanded from 96 care and treatment centres (CTC) in 2004 to 860 by 2011 (23), and currently, Tanzania has a total of 6206 facilities offering ART services where 2103 of them are CTC’s and 4103 are Option B+ facilities focusing on pregnant women to prevent mother to child transmission(PMTCT) as WHO guideline of 2015(Unpublished source – National Aids Control Programme(NACP). By 2018, 75% of all people living with HIV in Tanzania were estimated to be on treatment(24).

This paper describes trends in HIV prevalence and incidence in North-West Tanzania from 2006-2016 using Kisesa observational HIV cohort study data. ART services are provided from 5 dispensaries and one health centre. Eight rounds of sero-surveys, including HIV testing, have been conducted between 1994 and 2016, alongside 32 follow-up rounds of the Health and Demographic Sentinel Surveillance (HDSS). These data provide an unparalleled resource for understanding the HIV incidence trends in the population because, since 1994, the population has been under surveillance and has grown from 20,000 to 35,000 in 2016.

## Methods

### Study design

The Magu Health and Demographic Sentinel Surveillance study is located 20 kilometres East of Mwanza City-Tanzania(19). The study included a Kisesa observational HIV cohort with a nested series of cross-sectional sero-surveys measuring HIV Status, awareness of HIV, and access to regular HIV services among adult residents aged 15 years and above. Since 1994, eight community-based HIV sero-surveys have been conducted, with the data from the most recent sero-surveys in 2007, 2010, 2013 and 2016.

### Data and blood sample collection

After written informed consent, HDSS residents were interviewed by the same-sex interviewer using a standardised questionnaire and asked to give anonymous blood sample collected as dried blood spots (DBS).

To participate in the sero-survey, an individual must have been registered on the previous immediate HDSS follow-up listing. A unique identifier number was used to link the blood samples and sero-survey questionnaire.

Voluntary counselling and testing (VCT) for HIV has been offered since 2004, using the current national HIV guidelines at each time point (25,26). All DBS samples were tested by ELISA in the National Institute for Medical Research (NIMR) Mwanza laboratory by Uniform II Category III Ab test followed by Enzygnost test (sero-survey 5 and 6), a Uniform II Category IV Ab+Ag test followed by Enzygnost test (sero-survey 7) and Uniform II Vironostika HIV1/HIV2 for screening and Uniform II Enzygnost HIV1/HIV2 for confirmatory (sero-survey 8). In the 8^th^ sero-survey, the VCT results were used to diagnose HIV infection, and participants were able to opt-out of receiving the result if they did not want to know their HIV status. In all sero-surveys, quality control (5%) for HIV testing was provided by the NIMR laboratory in Mwanza City.

### Statistical analyses

Data from HDSS round 20(2007), 24(2010), 27(2013) and 32(2016) were linked with the sero-surveys carried out in the same year (Sero 5, 6, 7 and 8). Details of the sero-surveys are available elsewhere(19). HIV prevalence was estimated as the proportion of resident survey participants who tested positive for HIV in each survey round, weighted from the overall population.

Also, by combining the HIV prevalence with the proportion of individuals accessing antiretroviral therapy (ART), we produced the prevalence of untreated HIV, which is used as a factor explaining HIV incidence in the cohort. The untreated prevalence in potential heterosexual partners was derived from the partner’s age range, proportion of HIV positive individuals at each study round and the proportion of ART-naïve HIV-positive person-years. The partner age range was based on an age mixing matrix derived from the reported ages of sexual partners, and we used the 5^th^ and 95^th^ percentiles of this distribution to define the range within which these two proportions were estimated. The partner age range was calculated for everyone and was age-, sex-, and period-specific.

The HIV incidence analysis included all initially HIV negative residents who had at least one subsequent HIV test result. People entered the analysis at the date of their first HIV negative test and contributed person-time until the date of their last negative test or if they seroconverted. Participants who out migrated and later returned to the study contributed data only when they were residents in the study area.

The seroconversion per 1000 person-years and the incidence rates were calculated using Poisson regression separately for men and women in three calendar periods: 2006-2009, 2010-2012, and 2013-2016. Those who tested negative prior to 2006 entered observation in 2006. ART-naïve HIV-positive person-years were calculated to be HIV person-years before any recording of ART initiation. Person time at risk was calculated as the time between the HIV tests. HIV seroconversion was observed to have occurred in the interval between the last HIV negative test and the first HIV positive test date, with no limit on the length of the seroconversion interval. Those who remained HIV negative were censored from the analysis at the last HIV negative test date. Exit of the participant was out-migration from the study area, death and administrative censoring at the most recent study round, which was the last HIV test.

Risk factor analyses were conducted with time-varying exposure variables to account for time and changes in reported demographic characteristics and sexual behaviour reported in HDSS and serosurveys. The time variables included age, split into two age groups (15-24 years and 25-49 years), and three intervals (2006-2009, 2010-2012 and 2013-2016). Demographic variables included marital status, residence, education level, residential mobility (moved house in the last 12 months or not). Sexual behaviour variables included having a casual partner in the past year and the number of sexual partners in the previous year.

Cox regression models were fitted to estimate the hazard ratios with their associated 95% CI to explore the association between the variables listed above and HIV incidence. In addition, we fitted two adjusted models by sex, the first one adjusting for age categories as our potential confounder (Model 1) and the second model adjusted for age categories and other variables that showed a significant association with HIV incidence on crude analysis and significant results in model 1.

### Study Results

#### HIV Prevalence

The numbers of participants aged 15 years and above in the cohort prior to each of the sero-surveys were: 17697 in 2006 (Sero 5), 17336 in 2010 (Sero 6), 17774 in 2013 (Sero 7) and 18659 (Sero 8) in 2016. Of these 8,687 (49%) attended the serosurvey in 2007, 7964 (46%) in 2010, 7580 (43%) in 2013, and 7424 (40%) in 2016. The overall HIV prevalence decreased from 7.2% (in 2006), to 6.5% (2010), 7.0% (2013) and 6.6% in 2016. The crude HIV prevalence with its 95% confidence interval, stratified by age and sex in Kisesa at each time point, is shown in Table 1.

The prevalence of HIV increased among the older age groups (35+), although this was not significant during the period 2006 to 2010 (P-value >0.05), and we had a more considerable prevalence increase in women than in men. Conversely, the prevalence among younger age groups went down over the years, with almost a 50% reduction among men and women aged 15-24 years and a 33% reduction among both sexes aged 25-34 years (Figure 1).

Among the 489 HIV positive seen during 2016/17 (serosurvey 8), the self-reported access to care is shown in Table 2. Overall, 140 (29%) reported never having an HIV test before the sero-survey. A further 35 (7%) had tested for HIV but reported not accessing the HIV care services. Thus, a total of 230 (47%) reported attendance at the HIV care services but not on ART, of whom 80(16%) reported initiating ART.

#### HIV Incidence

Between 2006 and 2016, there was 59 seroconversion for men and 119 for women equivalent to 12911 person-years risk for men and 21590 for women. The incidence rate was estimated to be 4.57 per 1000 person-years in men, whereas the rate for women was 5.51, with an overall incidence rate of 5.14 per 1000 person-years (Table 3).

There was no clear trend in the incidence rate over time; however, we had a lower incidence rate estimate of 4.15 in 2013-2016 compared to 5.59 per 1000 person-years in 2006-2009 in women; a similar trend is observed in males with the incidence rates of 3.85 in 2013-2016 compared to 4.72 per 1000 person-years in 2006-2009. In addition, higher incidence rates were observed in the younger age group (15-24years) than older ones (25-44years).

We obtained a higher incidence rate among men who were either widowed or separated at 6.52 compared to the never-married 5.71 and married men 3.62 per 1000 person-years. This was different for women; we estimated a higher incidence rate on married group 6.03 compared to never-married 4.04 per 1000 person-years and separated or widowed group 3.94 per 1000 person-years. We had a much lower incidence rate for both genders on residents who completed secondary education or higher than lower levels; however, most of the differences we observed were not statistically significantly different.

### Factors associated with HIV Incidence

To determine the factors associated with the incidence of HIV, we estimated the hazard ratios by sex presented in Tables 4 and 5. On crude analysis, the number of sexual partners and prevalence of untreated HIV in the opposite sex were associated with the incidence of HIV in males, while in women, marital status and mobility were associated with the incidence of HIV.

Men who reported to have two or more sexual partners in the last year had five times higher hazard ratio of HIV incidence (HR=5.76 95% CI: 1.03-34.01) compared of those reported having 0 partner, while for men, a unit percentage increase of untreated HIV prevalence across surveys in potential heterosexual partners increased the hazard of acquiring HIV infection by 3% (HR=1.03 95% CI:1.01-1.21). Married women had a higher hazard rate (HR=1.98 95% CI:1.03-3.72) than unmarried and divorced or separated groups; also, mobile women had two times more hazards of HIV incidence (HR=2.35 95% CI:1.01-5.53) than non-mobile women.

In our adjusted models with age categories only, we obtained a higher hazard rate of more than three times for both married (HR=3.99 95% CI:1.31-12.23) and divorced or separated men (HR=5.34 95% CI:1.22-23.34) compared to unmarried men. In women, after adjusting for age groups, married (HR=2.73 95% CI:1.23-6.18) and mobile women had two times the higher hazard of HIV infection (HR=2.29 95% CI:1.01-5.43) compared to non-mobile women. None of the variables showed a significant association with HIV incidence for either gender in the final models.

## Discussion

Overall, our study results have shown remarkable progress in reducing both HIV prevalence and incidence in the Magu HDSS area. There was a significant decrease in HIV prevalence in the youngest age groups <24, from 8.3% to 6.5% between 1994 and 2000(18), and 1.5% in 2007 to 0.6% in 2016 in men and 4.5% to 2.0% in women. The estimated prevalence on the current sero-survey was 6.6% which was higher compared to the current national HIV prevalence in Tanzania among people aged 15 and above, which is 4.9%, and for Mwanza, 5% among men and 9.4% for women, which is higher compared with our estimate (20).

This cohort estimated a higher HIV prevalence for both men and women aged 25-55 years than younger ages of less than 25 and older ages above 55, which is the same trend observed in national estimates (20,27). This increase was expected due to better survival among HIV infected people, thanks to the increased ART availability and uptake. Data from rural South Africa and many other sub-Saharan countries have shown a similar trend (28,29). Although this decrease is significant, women are still more infected than men despite the availability of several preventive options like condom use, male circumcision programs, knowledge of HIV Status and HIV treatment to prevent further transmission. Unfortunately, most of these methods are male-based and provide fewer options to women(30,31).

The incidence of HIV remained high in all age groups, both in men and women in this cohort study, despite ART availability. The highest incidence rates in men are in the 35-to-54-year age groups, whereas the highest rates in women were in the age group 25-44 years. Older men prefer having a sexual relationship with younger women who are at higher risk than men of the same age. Also, the uptake of ART services is higher in older people (both men and women) than younger people, thus greater transmission opportunity to males with younger sexual partners. However, these results are not so much different from the reported data from the same cohort by Mwita et al., which showed the incidence increase in men as age increases and in other studies in Africa (1,4,5,18,32).

The fact that the incidence of HIV remained high despite the availability of ART is surprising but must be considered in light of the changes in ART national guidelines over the years explored by the four sero-surveys. Voluntary counselling and testing (VCT) for HIV have been offered since May 2005 in Kisesa health centre, with HIV testing and counselling (HTC) now available in all health facilities serving the cohort population. Nationally, ART has been available in Tanzania since 2004 and locally accessible from the zonal referral hospital in Mwanza since 2005 and Kisesa health centre since August 2008. However, until 2010 the criteria for ART initiation were a CD4 count ≤200 cells/mm3 or a WHO clinical stage of 4 for all adults. From 2010 to 2012, the criterion was CD4 count ≤350 cells/mm3. From 2013 to 2015, it changed to CD4 count ≤500 cells/mm3 and finally, since 2016, WHO’s Test and Treat Policy(T&T) was introduced (33–36). The slow lowering of thresholds to initiate ART and the progressive rolling out of ART in the country since the last guideline probably explains why the widespread availability of ART has not impacted HIV incidence yet.

Among the factors assessed, marital status (married or previously married), mobile individuals with more than one sexual partner were found to be associated with increased incidence of HIV. Since this is an open cohort, we would expect more provision of education on HIV prevention and change of risky sexual behaviour, which would have led to a reduction in the new HIV cases.

### Limitation

Due to missing data, some potential exposure variables like condom use and other sexual risk behaviour were not assessed to determine their association with the HIV infection rate.

In our study, the overall response rate of the eligible individual was less than 50% and much higher in women than in men. Hence the reported HIV prevalence and incidence may be subjected to bias due to non-response. Further analysis accounting for missing data will be conducted and reported in a different paper.

## Conclusion

Findings from our study show that HIV is still a problem in Tanzania. Although the prevalence of HIV is decreasing, the incidence is still high, indicating that we still have new infections in our community and far away from the HIV elimination level. The 90-90-90 UNAIDS target was not met by 2016, with new HIV guidelines and 95-95-95 goal, both prevalence and incidence must be assessed to evaluate the impact of HIV/AIDS programmes in Tanzania. Despite the wide availability of ART services, uptake and adherence to treatment of HIV/AIDS remain a big problem. Monitoring and supporting other interventions programs in the community will be another way of bringing down the new HIV infections, especially in these areas where resources are limited.

## Figures and Tables

**Table 1:** The prevalence of HIV by age and sex in residents of the Kisesa observational HIV cohort, 2006 to 2016

Characteristic	2006			2010			2013			
Age	Total seen	HIV +ve	Prevalence of HIV (95% CI)	Total seen	HIV +ve	Prevalence of HIV (95% CI)	Total seen	HIV +ve	Prevalence of HIV (95% CI)	Total seen
**Overall**	8687	622	7.2 (6.6, 7.7)	7964	516	6.5 (5.9,7.0)	7580	534	7.0 (6.4,7.6)	7424
**Males**										
15-24	1581	24	1.5 (0.9, 2.1)	1498	11	0.7 (0.2, 1,1)	1305	6	0.5 (0.1, 0.9)	1193
25-34	696	63	9.1 (6.9, 11.2)	457	36	7.9 (5.4, 10.4)	440	38	8.6 (6.0, 11.2)	400
35-44	489	56	11.5 (8.6, 14.3)	411	51	12.4 (9.2, 15.6)	391	49	12.5 (9.2, 15.8)	409
45-54	355	41	11.5 (8.2, 14.8)	307	39	12.7 (8.9, 16.4)	326	39	12.0 (8.4, 15.5)	330
55+	497	31	6.2 (4.0, 8.3)	426	22	5.2 (3.1, 7.3)	450	33	7.3 (4.9, 9.7)	455
**Females**										
15-24	1704	77	4.5 (3.5, 5.5)	1724	56	3.2 (2.3, 4.0)	1473	30	2.0 (1.3, 2.7)	1514
25-34	1266	156	12.3 (10.5,14.1)	1121	126	11.2 (9.3, 13.1)	1072	134	12.5 (10.5, 14.5)	1001
35-44	808	98	12.1 (9.8, 14.3)	807	104	12.9 (10.5, 15.2)	798	114	14.3 (11.8, 16.7)	791
45-54	546	48	8.8 (6.4, 11.2)	510	49	9.6 (7.0,12.2)	551	54	9.8 (7.3, 12.2)	552
55+	745	28	3.8 (2.4, 5.2)	703	22	3.1 (1.8, 4.4)	774	37	4.8 (3.3, 6.3)	779

**Table 2:** Self-reported access to HIV services by HIV positive residents of the Kisesa cohort seen in 2016, by age and sex.

Characteristic	2016 (sero 8)			
Age	Total HIV positive	Undiagnosed (did not know their HIV status)	Diagnosed but not enrolled in care	Enrolled in care, but not on ART
**Males**				
15-24	7	3	1	3
25-34	25	11	4	8
35-44	52	15	1	30
45-54	33	10	2	15
55+	31	7	1	16
**Females**				
15-24	31	9	4	18
25-34	85	22	6	50
35-44	103	21	9	46
45-54	68	18	5	27
55+	50	24	2	17
**Total**	485	140	35	230

• Four HIV (2 males and 2 females) missing data on HIV status

**Table 3:** HIV Incidence rates in males and females from 2007 to 2016 in Kisesa, Tanzania

	Males				
Characteristic	No. of sero-conversions	Average Person-years at risk	HIV incidence Rate (95% CI) per 1000 pyrs	No. of sero-conversions	Averag years
**Overall**	59	12911	4.57 (3.54, 5.89)	119	21590
**Age**					
15-24	18	4776	3.77 (2.37, 5.98)	25	4571
25-34	8	1734	4.61 (2.31, 9.22)	36	4862
35-44	14	1906	7.34 (4.35, 12.40)	30	4100
45-54	13	1850	7.03 (4.08, 12.10)	16	3316
55+	6	2644	2.27 (1.02, 5.05)	12	4741
**Period**					
2007-2009	21	4453	4.72 (3.07, 7.23)	40	7154
2010-2012	24	4825	4.97 (3.33,7.42)	52	7937
2013-2016	14	3633	3.85 (2.28, 6.51)	27	6499
**Residence**					
Rural	41	8581	4.78 (3.51, 6.49)	69	12646
Peri-urban	12	2402	4.99 (2.84, 8.79)	30	4474
Urban	6	1928	3.11 (1.39, 6.93)	20	4500
**Marital status**					
Never married	16	4418	3.62 (2.23, 5.91)	11	2721
Married	38	6656	5.71 (4.15, 7.85)	75	12430
Widowed/Separated	4	614	6.52 (2.25, 17.37)	19	4820
**Education**					
No formal	10	2409	4.15 (2.23, 7.71)	51	9052
Incomplete primary	13	2640	4.93 (2.86, 8.48)	18	2774
Completed primary	29	6272	4.62 (3.21, 6.65)	47	8678
Completed secondary and Above secondary	3	815	3.68 (1.19, 11.42)	1	589

**Table 4:** Hazard rate ratios for HIV incidence among male residents in Kisesa Open HIV cohort.

Characteristics	Crude Hazard Ratio (95% CI)	Age-Adjusted Hazard Ratio (95% CI)+	Final model (95% CI) ++
**Age groups**			
15-24	0.79 (0.47-1.35)	0.79 (0.47-1.35)	1.74 (0.68-4.45)
25+	1	1	1
**Period**			
2007-2009	1	1	
2010-2013	0.83 (0.39-1.77)	0.84 (0.39-1.80)	
2013-2016	0.51 (0.19-1.40)	0.52 (0.19-1.43)	
**Residence**			
Urban	1	1	
Peri-Urban	1.62 (0.68- 3.82)	1.60 (0.68-3.77)	
Rural	1.64 (0.62 - 4.38)	1.62 (0.60-4.31)	
**Marital Status**			
Never married	1	1	1
Married	1.55 (0.86-2.81)	3.99 (1.31-12.23)	0.64(0.03-9.13)
Widowed/Separated	1.77 (0.59-5.30)	5.34 (1.22-23.34)	1.21(0.04-25.12)
**Education level**			
No formal education	1	1	
Incomplete primary	1.15 (0.50-2.63)	1.16 (0.50-2.72)	
Completed primary	1.11 (0.54-2.28)	0.86 (0.41-1.83)	
Completed secondary/Higher	0.90 (0.25-3.28)	0.78 (0.24-3.28)	
**Mobility**			
Non-Mobile	1	1	
Mobile	1.48 (0.44-5.61)	1.18 (0.33-3.92)	
**Casual partner**			
None	1	1	
One or more	1.09 (0.28-4.62)	1.18 (0.45-4.85)	
**Number of sexual partners in last year**			
None	1	1	1
One	3.89 (0.78-20.02)	2.46 (0.47-13.08)	1.24 (0.17-5.35)
Two or more	5.76 (1.03-34.01)	3.35 (0.52-18.42)	3.02 (0.48-18.78)
Prevalence of untreated HIV infections (Per unit percentage increase)	1.03 (1.01-1.21)	1.01 (0.68-1.53)	1.06 (0.65-1.49)

+ Adjusted for age in five age groups – 15-24, 25-34, 35-44, 45-54, 55+ years.

++ Adjusted for age groups, significant factors independently associated with HIV incidence or significant in +.

**Table 5:** Hazard rate ratios for HIV incidence among female residents in Kisesa Open HIV cohort.

Characteristics	Crude Hazard Ratio (95% CI)	Age-Adjusted Hazard Ratio (95% CI)+
**Age groups**		
15-24	1.24 (0.86-1.79)	1.24 (0.86-1.79)
25+	1	1
**Period**		
2007-2009	1	1
2010-2013	0.84 (0.50-1.40)	1.16 (0.70-1.91)
2013-2016	0.74 (0.38-1.43)	1.00 (0.56-1.81)
**Residence**		
Urban	1	1
Peri-Urban	1.29 (0.78-2.12)	1.23 (0.75-2.02)
Rural	1.53 (0.87-2.70)	1.49 (0.84-2.62)
**Marital Status**		
Never married	1	1
Married	1.98 (1.03-3.72)	2.73 (1.23-6.18)
Widowed/Separated	1.19 (0.58-2.46)	1.52 (0.61-3.57)
**Education level**		
No formal education	1	1
Incomplete primary	1.10 (0.64-1.89)	0.89 (0.51-1.55)
Completed primary	0.92 (0.62-1.37)	0.66 (0.43-1.02)
Completed secondary/Higher	0.29 (0.04-2.10)	0.23 (0.03-1.65)
**Mobility**		
Non-Mobile	1	1
Mobile	2.35 (1.01-5.53)	2.29 (1.01-5.43)
**Casual partner**		
None	1	1
One or more	1.53 (0.34-6.67)	1.62 (0.37-7.03)
**Number of sexual partners in last year**		
None	1	1
One	1.41 (0.39-4.95)	1.31 (0.33-5.21)
Two or more	2.23 (0.76-6.83)	2.09 (0.57-6.98)
**Prevalence of untreated HIV infections (Per unit percentage increase)**	1.13 (0.76-1.21)	1.15 (0.75-1.38)

+ Adjusted for age in five age groups – 15-24, 25-34, 35-44, 45-54, 55+ years.

++ Adjusted for age groups,significant factors independently associated with HIV incidence or significant in +.

## Data Availability

Dataset used in the analysis will be made available from the corresponding author on reasonable request.
